# Antioxidant and Anticholinesterase Properties of the Aqueous Extract of *Balanites aegyptiaca* L. Delile Fruit Pulp on Monosodium Glutamate-Induced Excitotoxicity in Swiss Mice

**DOI:** 10.1155/2022/7576132

**Published:** 2022-04-11

**Authors:** Bouvourné Parfait, Beppe Galba Jean, Ponka Roger, Ngatanko Abaissou Hervé Hervé, Kamleu Kwingwa Balbine, Camdi Woumitna Guillaume, Guedang Nyayi Simon Desire, Damo Kamda Jorelle Linda, Kenko Djoumessie Léa Blondelle, Sotoing Taiwe Germain

**Affiliations:** ^1^Department of Biological Sciences, Faculty of Science, University of Maroua, P.O. Box 814, Maroua, Cameroon; ^2^Department of Agriculture, Animal Husbandry and By-Products, National Polytechnique School, University of Maroua, P.O. Box 46, Maroua, Cameroon; ^3^University of Buea, Department of Zoology and Animal Physiology, Faculty of Science, P.O. Box 63, Buea, Cameroon

## Abstract

*Balanites aegyptiaca* L. Delile (*B*. *aegyptiaca*) is used in traditional medicine for the treatment of memory impairment. This work aims to evaluate the antioxidant and anticholinesterase potential of BA fruit pulp extract on excitotoxicity induced by monosodium glutamate (MSG). MSG was administered 30 minutes after treatment with *B*. *aegyptiaca* aqueous fruit pulp extract (50, 125, 250, and 500 mg/kg) and vitamin C (100 mg/kg) for 30 days. The negative control group received only MSG, while the control group was given distilled water daily. Behavioral tests parameters (using the novel object recognition, Y-maze, and Barnes maze tests), oxidative stress biomarkers (malondialdehyde, superoxide dismutase, and catalase), nitric oxide, and acetylcholinesterase activity and hippocampal architecture were evaluated. Results obtained revealed that different doses of *B*. *aegyptiaca* significantly reversed the deleterious effect of MSG on memory. This was displayed by a significant (*p* < 0.05) increment in the percentage of spontaneous alternation in the Y-maze test and a significant (*p* < 0.001) increase in discrimination index in novel object recognition observed with 500 mg/kg extract dose. Moreover, the extract (250 and 500 mg/kg doses) significantly (*p* < 0.001) increased direct search strategy and significantly decreased (*p* < 0.01) the time taken to find the target hole in the Barnes maze. A modulation of hyperactivity was observed after administration of all extract doses compared to the negative control group in the open arena. Furthermore, the highest dose of the extract caused a significant (*p* < 0.001) improvement in antioxidant enzymes activity, associated with a significant (*p* < 0.001) decrement in nitric oxide and malondialdehyde concentrations and a significant (*p* < 0.01) decrease in acetylcholinesterase activity. Treatment with the extract also restored normal hippocampal cell architecture. *B*. *aegyptiaca* fruit pulp extract could thus confer neuroprotection through its antioxidant and anticholinesterase potential.

## 1. Introduction

Alzheimer's disease (AD) is a progressive, irreversible, and incurable affection characterized by behavioral changes, cognitive, and functional deficits [1]. It represents the main cause of dementia syndrome, accounting for at least 70% of cases [2]. Cognitive symptoms of this disease include mainly deficits in short-term memory, praxis, visuospatial, and executive dysfunction [3]. Epidemiological studies revealed that approximately 36 million people worldwide suffer from AD, and this is expected to reach 115 million by 2050 if no effective therapy is discovered [4]. The increase in life expectancy nowadays even makes this disease a growing public health challenge [5]. However, many questions about the pathogenesis of this devastating disease remain unanswered and with very few existent satisfactory treatment options. Besides old age, genetic predisposition and stress are factors that may contribute to AD-associated disorders such as memory loss, learning disabilities, and dementia [6]. *β*-amyloid protein aggregation, tau protein hyper-phosphorylation, acetylcholine (ACh) reduction, and glutamatergic deficiency are considered as main hallmarks of AD [7]. Recent studies have hypothesized that free radicals produced during oxidative stress and/or inflammatory processes are also important from a pathological point of view [8]. In order to understand Alzheimer's type memory loss, many animal models using scopolamine, L-arginine, and monosodium glutamate as inducers have been developed. Monosodium glutamate (MSG), a sodium salt of L-glutamic acid, has been used for several years as a taste enhancer [9]. MSG administration has been shown to increase hippocampal glutamate (Glu) levels, thereby significantly altering neurobehavioral performance in rats, causing anxiety and memory impairment [10, 11]. According to Rajagopal et al. [12], the excitotoxic and the oxidative pathways are two distinct routes by which glutamate induces neuronal death. Sustained high concentration of MSG in the synaptic cleft leads to excessive activation of glutamate receptors and subsequent necrosis [13, 14], by increased calcium entry, oxidative stress with the free radical generation, and mitochondrial dysfunction, which can cause apoptosis [15]. However, the molecular mechanisms of MSG-induced learning and memory impairment may be related to the alteration of certain neurotransmitters levels and the activity of enzymes related to neurotransmitter metabolism, such as acetylcholinesterase (AChE) [16] resulting in poor cognitive function. Acetylcholinesterase (AChE) are key enzymes that cause the degradation of acetylcholine (Ach) resulting in cholinergic deficits and termination of cholinergic neurotransmission [17]. Acetylcholine (ACh) and butyrylcholine (BCh) are important neurotransmitters that are involved in the acquisition and storage of memories and in the transmission of impulses across a synapse [18]. The existing therapy of this devastating pathology (AD) is based on the use of acetylcholinesterase inhibitors such as donepezil and rivastigmine [19] and NMDA receptor antagonists, such as memantine. These chemically formulated drugs used cause undesirable side effects in patients [20]. Moreover, the inaccessibility of these drugs for some patients and the high cost for others remain major problems for the population [21, 22]. In the quest for more effective and innovative treatment strategies for the subsequent development of therapeutic compounds, herbal medicine is highly solicited. In the past, medicinal plants have been used in the treatment and prevention of several pathologies. Many papers reported the neuroprotective effects of fruits (especially berries) and vegetables, which contain nutrients and constitute rich sources of antioxidant compounds [23]. Antioxidants are chemical substances that preclude oxidation or reduce the levels of free radicals in human bodies [18]. Additionally, the search for natural antioxidants present in medicinal plants is very important due to their potential for extensive use in practical applications [24, 25]. According to Ngo Bum et al. [26] some of these plants have pharmaceutical properties by acting as placebos. Hence, their pharmacological activity needs to be highlighted through scientific studies in order to vulgarize their use. Many plants have shown neuroprotective properties, such as *Ziziphus mucronata* [27, 28]. This research work focuses on the valorization of *Balanites aegyptiaca* (Zygophyllaceae) commonly called desert date fruit pulp. It was reported in traditional medicine use in the treatment of anxiety, epilepsy, and mental disorders [29]. It has been reported as a strong laxative and abortifacient endowed to treat insanity diabetes, jaundice, yellow fever, syphilis, helminths infestation, cough, [30]. Furthermore, various extracts of this plant have been scientifically proven to possess anticonvulsants [31] and analgesic effects [32]. However, there is no published data on the neuroprotective effects of *B*. *aegyptiaca* fruit pulp found to date. This study was thus undertaken to evaluate the neuroprotective potential of *B*. *aegyptiaca* fruits pulp extract against oxidative stress, excitotoxicity, and neuronal damage induced by monosodium glutamate in Swiss mice.

## 2. Material and Methods

### 2.1. Plant Material and Extraction

The plant material consisted of *B*. *aegyptiaca* fruits, collected from Mayo-Oulo Division (Garoua, North region, Cameroon) in 2018. The plant was identified by Professor Tchopsala (Taxonomist) and authenticated at the Fauna School of Garoua by comparing it with a specimen 42148/HNC.

### 2.2. Preparation of *B*. *aegyptiaca* Fruit Pulp Extract

Fruit pulp was separated from the seed, dried, and pulverized into a fine powder. To achieve extraction, 70 g of the powder obtained was dissolved in 700 mL of distilled water for 24 hours (as per traditional healer), filtered, and then cooled to room temperature before being lyophilized. The lyophilizate obtained was used to prepare different extract doses.

### 2.3. Determination of Polyphenolic Compounds

Total phenolic, flavonoid, and tannins contents were quantified according to the methods of Singleton et al. [33], Braindbridge et al. [34], and Gaytan-Martínez et al. [35] respectively.

### 2.4. Animal Material

Thirty-five (35) male Swiss mice weighing 25 ± 5 g were used in this experiment. These animals were bred in the animal house of the laboratory of Biological Sciences of the University of Maroua. They were then acclimatized for two weeks in the Laboratory of Medicinal Plants, Health and Galenic Formulation of the University of Maroua before the beginning of the experiment. Mice were fed with a diet consisting of (50% corn flour, 10% fish powder, 10% bone powder, 10% cottonseed cake, 10% soya bean flour, 3% salt, 5% oil, and 2% water), given access to water ad libitum and maintained in a temperature and light-controlled room (25 ± 2°C, natural day/night cycle). Animals were handled according to the guidelines of the Cameroon Bioethics Committee (reg. no. FWA-IRB00001954). The protocol was approved by the ethics committee of the Faculty of Sciences of the University of Maroua (ref. no. 14/0261/Uma/D/FS/VD-RC).

### 2.5. Chemical Material

Glutamate monosodium, thiobarbituric acid (TBA), trichloroacetic acid (TCA), hydrogen peroxide (H_2_O_2_), and ascorbic acid were purchased from Sigma-Aldrich (St. Louis, MO, USA). Acetylthiocholine iodide and 5,50-dithiobis (2-nitrobenzoic acid) (Ellman reagent), ethanol, phosphoric acid, sodium hydroxide, anhydrous sodium, sodium bicarbonate, dichromate acetic acid, sodium citrate, formalin, and sodium pentobarbital were purchased from Biochemical (Shanghai, China). All drugs and extract were freshly prepared in saline on the day of the experiment.

### 2.6. Mice Treatment

The method used was described by Waggas [36]. Mice were distributed into seven groups (*n* = 5): a normal control group (distilled water only), a negative control group (4 mg/kg/day i.p. of MSG), four test groups (*B*. *aegyptiaca* pulp extract 50 mg/kg, 125 mg/kg, 250 mg/kg, and 500 mg/kg/day p.o. + 4 mg/kg/day i.p. of MSG). The positive control group was given vitamin C (100 mg/kg/kg/day p.o. + MSG at 4 mg/kg/day i.p. (Table 1). Treatment was administered for 30 days, and mice were subjected to a behavioral test battery 24 hours after the last administration.

Doses of *B*. aegyptiaca extract were determined as per the traditional healer (one glass per day for an adult) and after a screening on mice tradition.

### 2.7. Behavioral Assessment

#### 2.7.1. The Novel Objects Recognition Test

The object recognition test was performed according to the experimental protocol of El-Marasy et al. [37] and was used to evaluate recognition in mice. Each mouse was placed in the apparatus and allowed to freely explore the apparatus for 5 min. On the next day, during the “sample” trial (T1), two identical objects were placed in two opposite corners of the apparatus; an animal was placed again in the apparatus; and the exploration time of objects was recorded for 5 min. The mouse was returned to its home cage, and an intertrial interval of 24 h was given. On the last day of the test, the “choice” trial (T2) was performed 30 min following glutamate injection. In T2, a new object (*N*) replaced one of the objects that were presented in T1, and then the mouse exploration time of the familiar (*F*) and the new objects (*N*) was assessed for 5 min. Exploration was defined as follows: directing the nose towards the object at a distance of not more than 2 cm and/or touching the object with the nose. The arena and objects were thoroughly cleaned with 70% ethanol in order to eliminate residual odors. From this measure, a series of variables was then calculated: the total time spent in exploring the two identical objects in T1 and that spent in exploring the two different objects, *F* and *N* in T2. The discrimination index (DI) was calculated according to the following formula: DI = *N* − *F*/*N* + *F*.

#### 2.7.2. Y-Maze Test

The Y-maze apparatus consisted of a chamber with three arms labeled A, B, and C, symmetrically separated at 120°. Each mouse was placed in arm A of the Y-maze apparatus and allowed to explore all three arms for 5 minutes. Frequency of arm entries and alternations (consecutive navigations through the three arms, that is, ABC, CAB, or BCA but not BAB or ABA) were assessed in this test [38]. The percentage (%) of correct alternations (an index of memory performance) was calculated as follows:

(1) $$\begin{array}{c} \left(\%\right)\text{Alternation}=\frac{\text{Total number of alternation}}{\text{Number of visits}-2}\times 100. \end{array}$$

The maze was cleaned with 70% ethanol after each test to remove residual odor.

#### 2.7.3. Barnes Maze Test

The test involved placing an animal at the center of the maze in a circular box. After 10 seconds, the box was removed, and the animal was left free to search for the escape cavity. The test ended when the animal found the escape chamber or when the time allowed to locate the escape chamber had elapsed. The test was performed in 3 phases with repetition possibilities for some phases [39, 40]. During the habituation phase, the animal was placed near the target hole where it freely explores the platform for 120 seconds; after this time, if the animal does not enter the target hole, it is guided there by the experimenter and left there for 60 seconds before being placed back in its home cage. The acquisition phase started 24 hours after the habituation and lasted 3 days with 4 repetitions per day and 300 seconds of interphase. It involved placing the animal at the center of the maze in a circular box. 10 s later, the box was removed and the animal was left to freely search for the escape cavity within 120 seconds. After this time, if the animal did not enter the target hole, it was guided towards it by the experimenter. Once in the target hole, it was left for 60 seconds, before being returned to its home cage. As for the retention phase, it started 24 hours after the acquisition and lasted 1 day only without any repetition. It also involved placing the animal in the center of the maze in a circular box 10 s later; the box was removed; and the animal was left to freely search the escape cavity during 120 s. The maze was thoroughly cleansed after each animal's passage to prevent the detection of the preceding mouse passage.

#### 2.7.4. Open-Field Task

The open-field test was used to assess both exploratory behavior and locomotor activity according to the protocol described by Bum et al. [41]. The test arena consisted of a box made from gray wood measuring 40 cm × 40 cm × 45 cm. The test consisted in placing an animal mouse at the center of the device and allowing it to freely explore the arena. The number of rearing, crossing, grooming, and the time spent at the center were recorded over a 5 minutes period for each animal. At the end of each observation, the mouse was returned to its home cage, and the device was cleaned with 70% ethanol.

### 2.8. Brain Sample Preparation

On day 31 of the experiment, all mice were anesthetized (using sodium pentobarbital, 100 mg/kg b.w., i.p.) and quickly decapitated. The entire brain was harvested for biochemical studies. The brain tissue samples were then homogenized with 0.1 M phosphate buffer (pH 7.4). The homogenate was centrifuged (3,000 rpm for 15 minutes at 4°C), and the supernatant was collected and stored at −20°C for biochemical assay.

#### 2.8.1. Determination of Malondialdehyde (MDA)

The protocol used was that described by Wilbur et al. [42]. The presence of malondialdehyde (MDA) in a sample results in the formation of malonic aldehyde in an acidic medium (pH 6.7) at a high temperature (100°C). The latter reacts with thiobarbituric acid to form a pink complex. The absorbance was read with a spectrophotometer at 530 nm against a blank. In the test tubes and the blank tube, 500 *μ*l of the homogenate and 250 *μ*l of Tris-HCl buffer (50 mM; KCl 150 mM; pH 7.4) were introduced. To each tube, 250 *μ*l of TCA (20%) and 500 *μ*l of thiobarbituric acid (0.67%) were added. The tubes were stopped with marble balls, incubated (10 min at 90°C), and cooled with tap water. These tubes were then centrifuged at 3,000 rpm for 15 min at room temperature. The supernatant was pipette, and the absorbance was read at 530 nm using a spectrophotometer against the blank.

#### 2.8.2. Nitrite Concentration

The accumulation of nitrite in the supernatant, an indicator of the production of nitric oxide (NO), was estimated using the principle of Griess reaction [43]. Brieﬂy, equal volumes of tissue homogenate and Griess reagent (5% phosphoric acid containing 0.1% NEDD (N,N-(1-naphthyl)ethylenediamine dihydrochloride), 1% sulfanilamide) were mixed and incubated in the dark for 10 min at room temperature. Then the absorbance of the reaction mixture was determined at 540 nm. The concentration of nitrite in the supernatant was determined from a sodium nitrite (NaNO_2_) standard curve. The amount of nitrite was expressed as nmol/g tissue.

#### 2.8.3. Determination of Superoxide Dismutase Activity

Superoxide dismutase (SOD) activity was determined according to the method described by Mishra and Fridovich [44]. The supernatant (140 *μ*L) was added to 1,660 *μ*L of carbonate buffer (pH = 10.2) and 200 *μ*L of freshly prepared adrenaline (0.3 mM). The change in optical density/minute was measured at 480 nm against the reagent blank. SOD activity was expressed as units/mg protein. The blank consisted of 140 *μ*l of distilled water, 1,660 *μ*L of carbonate buffer, and 200 *μ*L of adrenaline solutions.

#### 2.8.4. Determination of Catalase Activity (CAT)

Catalase activity was determined according to the method of Aebi et al. [45]. Twenty-five microliters of supernatant were added to 375 *μ*L of phosphate buffer. The reaction was started by the addition of 100 *μ*l of H_2_O_2_ (50 mM) and incubated at 37°C for 1 min, and the reaction was stopped by the addition of 1,000 *μ*l of dichromate prepared in 1% glacial acetic acid reagent. The tubes were then incubated for 10 min in a boiling water bath and cooled with tap water, and the absorbance was read at 570 nm. Catalase activity in the samples was obtained from a previously established calibration curve. Catalase activity was expressed as micromoles of H_2_O_2_/min/mg protein.

#### 2.8.5. Determination of Reduced Glutathione (GSH) Concentration

The determination of reduced glutathione (GSH) was performed according to the method of Ellman et al. [46]. 2,2-dithio-5,5′-dinitrobenzoic acid (DTNB) reacts with the SH groups of the glutathione present in the homogenate. This biochemical reaction gave a yellow-colored complex. The absorbance was read with a spectrophotometer at 412 nm against the blank. A volume of 1,500 *μ*l of Ellman's reagent was introduced into tubes containing 100 *μ*l of homogenate (test tube) and 100 *μ*l of tris-HCl buffer (blank tube). The resulting mixtures were incubated for 1 h at room temperature. The absorbance was read with a spectrophotometer at 412 nm against the blank.

#### 2.8.6. Estimation of Brain Acetylcholinesterase (AChE) Activities

The quantities of acetylcholinesterase were estimated by the method described by Ellman et al. [46]. For the estimation of acetylcholinesterase, 20 *μ*L of buffer Tris-HCl 0.1 M (pH 8.0) and 3 mL Ellman reagent were introduced into all tubes (test and blank vials) and then 100 *μ*L homogenate followed by 100 *μ*L Tris buffer (HCl 50 mM; KCl 150 mM; pH 7.4) in the blank tube. Subsequently, 20 *μ*L of 30 mM acetylthiocholine iodide were added to all the tubes. After a rapid homogenization of the mixture at room temperature, the absorbance was read at 412 nm after 30 s and 90 s against the blank.

### 2.9. Statistical Analysis

All results were expressed as mean ± standard error mean (SEM) and analyzed using Graph Pad Prism version 8.01 software. Data analysis was performed using one-way analysis of variance (ANOVA) followed by Bonferroni post-test. A significant difference was considered at *p* < 0.05.

## 3. Results

### 3.1. Polyphenolic Compounds of *B. aegyptiaca* Fruit Pulp

Table 2 shows the bioactive compounds of the *B. aegyptiaca* fruits pulp extract.

The results revealed the presence of several secondary metabolites components (polyphenols, flavonoids, and tannin). The contents of *B. aegyptiaca* pulp fruit in total polyphenols, total flavonoids, and total tanins were 78.13 ± 0.70 mg EGA/100 g DW, 48.73 ± 0.05 mg EQ/100 g DW, and 19.23 ± 0.06 mg EC/100 g DW, respectively.

### 3.2. Effect of the Aqueous Extract of *B. aegypriaca* Fruit Pulp on Short-Term Memory in the Y-Maze

**Figure 1:**
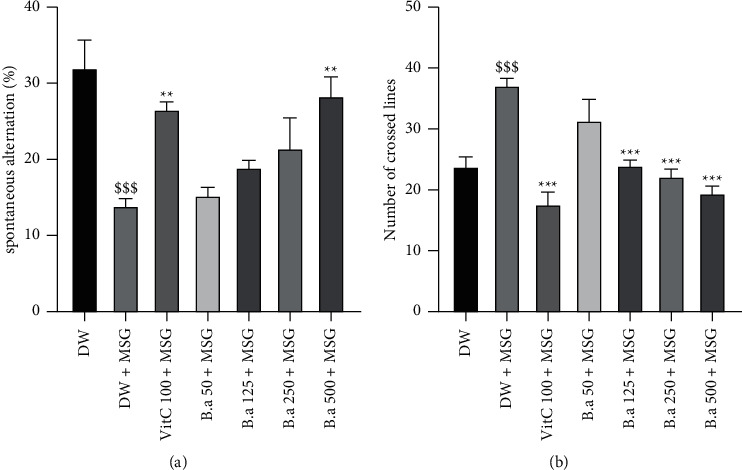
Effect of the aqueous extract of *B. aegyptiaca* fruit pulp on short-term memory in the Y-maze. Each column represents the mean ± SEM of six animals. (a) Percentage of alternation and (b) number of lines crossed. MSG: monosodium glutamate, Vit C: vitamin C, and DW: distilled water. ^*∗∗*^*p* < 0.01 and ^*∗∗∗*^*p* < 0.001 versus negative group. ^$$^*p* < 0.01 and ^$$$^*p* < 0.001 versus control group.

Figures 1(a) and 1(b) below show the effect of *B. aegyptiaca* fruit pulp extract on the percentage of spontaneous alternation and the number of lines crossed in the Y-maze, respectively. MSG administration resulted in a significant (*p* < 0.001) decrease in the percentage of spontaneous alternation compared to the normal control group. However, 500 mg/kg extract dose as well as vitamin C significantly (*p* < 0.01) increased the percentage of spontaneous alternation compared to the negative control group (Figure 1(a)). No significant difference was observed in the number of lines crossed after administration of various substances (Figure 1(b)).

### 3.3. Effect of the Aqueous Extract of *B. aegyptiaca* Fruit Pulp on Long-Term Memory in the Object Recognition Test

**Figure 2:**
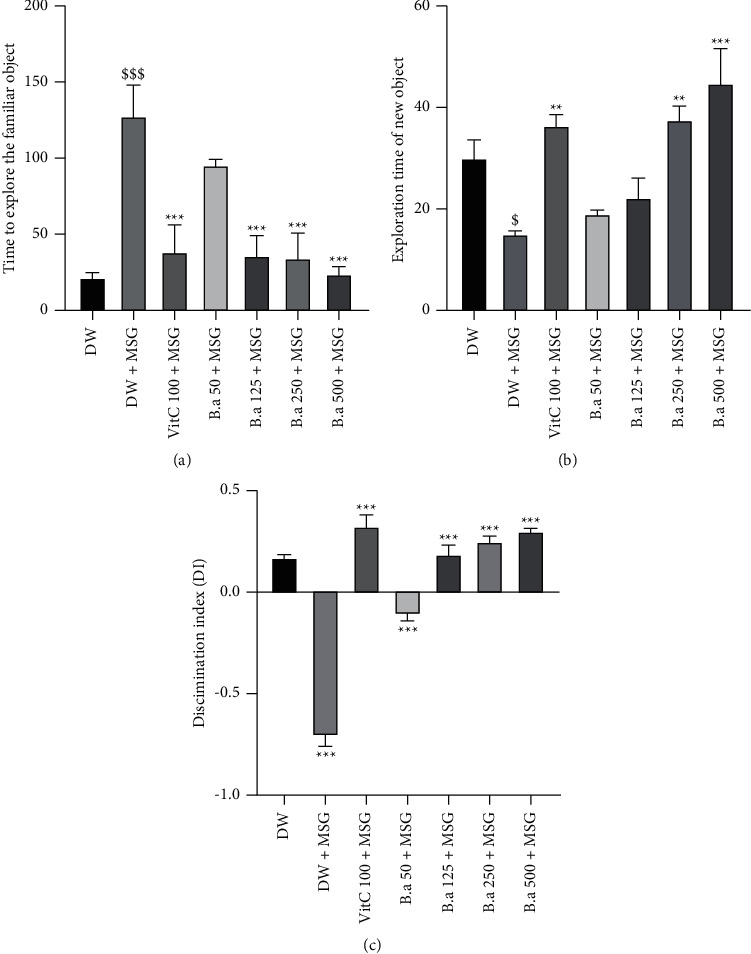
Effect of aqueous extract of *B. aegyptiaca* fruit pulp on long-term memory in the object recognition test. Each column represents the mean ± SEM of six animals. (a) Familiar object exploration time; (b) novel object exploration time; (c) discrimination index. MSG: monosodium glutamate, Vit C: vitamin C, and DW: distilled water. ^*∗∗*^*p* < 0.01 and ^*∗∗∗*^*p* < 0.001 versus negative group. ^$$^*p* < 0.01 and ^$^*p* < 0.01 versus control group.

Figure 2 below shows the effect of the aqueous extract of *B. aegyptiaca* fruit pulp on the exploration time of mice in the object recognition test. Mice treated with monosodium glutamate (negative control) solely showed a significant (*p* < 0.001) increase in familiar object exploration time compared to the normal control group. Pretreatment with the extract resulted in a significant (*p* < 0.001) decrease in familiar object exploration time at 125, 250, and 500 mg/kg doses compared to the negative control group (Figure 2(a)). On the other hand, a significant increase (*p* < 0.001) in the time taken to observe the new object was noted with the 500 mg/kg dose compared to the negative control group (Figure 2(b)). *B. aegyptiaca* fruit pulp (500 mg/kg) as well as vitamin C resulted in a significant (*p* < 0.001) increase in the time taken to observe the new object. Discrimination index (DI) was significantly (*p* < 0.001) diminished in MSG demented mice that received the vehicle as compared to normal mice. However, repeated treatment of mice with *B. aegyptiaca* at all doses as well as ascorbic acid (vitamin C) except the smallest dose induced an increase in DI (Figure 2(c)).

### 3.4. Effect of the Aqueous Extract of *B. aegyptiaca* Fruit Pulp on Spatial, Working, and Reference Memory in the Barnes Maze

**Figure 3:**
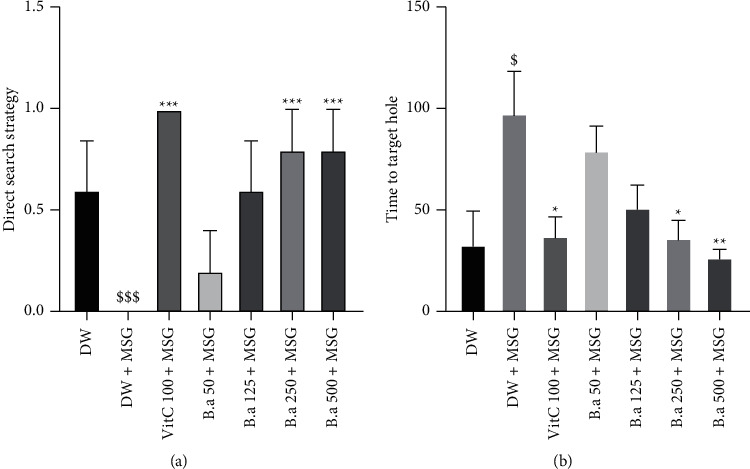
Effect of aqueous extract of *B. aegyptiaca* fruit pulp on spatial, working, and reference memory in the Barnes maze. Each column represents the mean ± SEM of six animals. (a)= Direct search strategy and (b) time to find the target hole. MSG: monosodium glutamate, Vit C: vitamin C, and DW: distilled water. ^*∗*^*p* < 0.05, ^*∗∗*^*p* < 0.01, and ^*∗∗∗*^*p* < 0.001 versus negative group. ^$^*p* < 0.05 and ^−^*p* < 0.001 versus control group.

Figures 3(a) and 3(b) show the direct search strategy and the time taken to find the target hole in the Barnes maze test are presented, respectively. Repeated administration of MSG (negative control) resulted in a significant (*p* < 0.001) increase in time taken to find the escape hole compared to the normal group. Administration of the aqueous extract of *B. aegyptiaca* fruit pulp (125, 250, and 500 mg/kg doses) resulted in a significant (*p* < 0.001) increase in direct search strategy compared to the negative control group that showed no direct search strategy (Figure 3(a)). The extract (250 mg/kg and 500 mg/kg dose) caused a significant decrease (*p* < 0.05 and *p* < 0.01, respectively) in the time taken to find the escape hole compared to the control group (Figure 3(b)).

### 3.5. Effect of the Aqueous Extract of *B. aegyptiaca* Fruit Pulp on Anxiety and Motor Coordination in the Open Field

**Figure 4:**
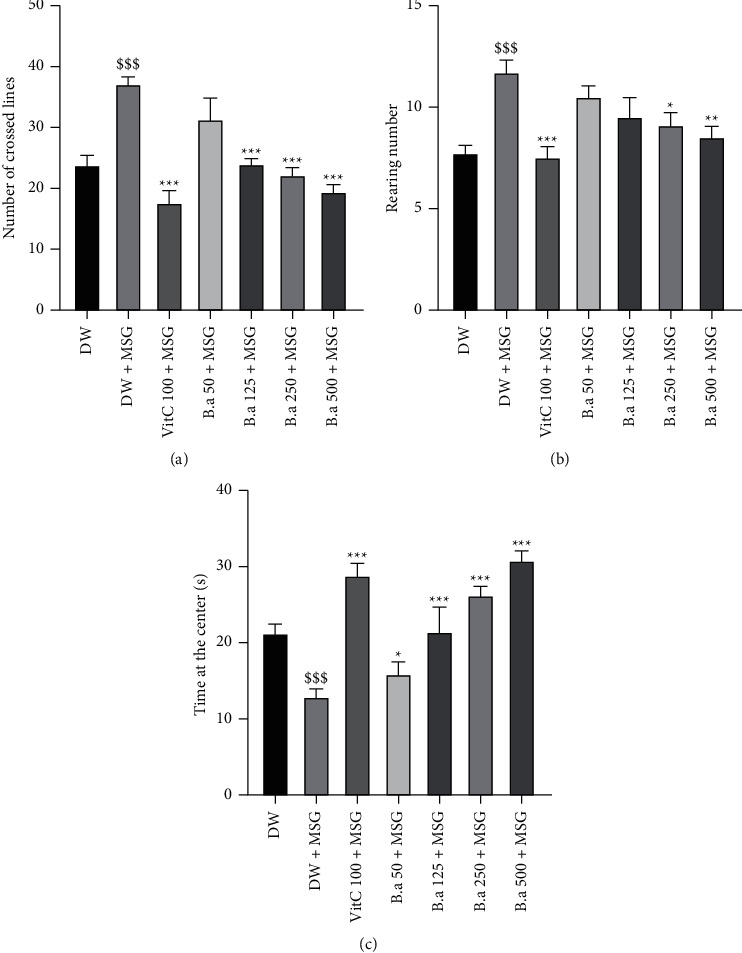
Effect of aqueous extract of *B. aegyptiaca* fruit pulp on anxiety and motor coordination in the open arena. Each column represents the mean ± SEM of six animals. (a) Numbers of lines crossed, (b) numbers of rearing, and (c) time spent in the center. MSG: monosodium glutamate, Vit C: vitamin C, and DW: distilled water. ^*∗∗*^*p* < 0.01 and ^*∗∗∗*^*p* < 0.01 versus negative group. ^$$^*p* < 0.05 and ^$$$^*p* < 0.001 versus control group.

Figure 4(a)–4(c) shows the number of lines crossed, the number of rearing, and the time spent at the center of the open field arena, respectively. Administration of monosodium glutamate solely resulted in a significant (*p* < 0.001) increase in the number of lines crossed and the number of rearing in the negative control group compared to the normal control group. Pretreatment with *B. aegyptiaca* fruit pulp extract resulted in a significant (*p* < 0.001) decrease in the number of lines crossed at 125, 250, and 500 mg/kg doses, compared to the negative control group (Figures 4(a) and 4(b)). Similarly, administration of the extract (250 mg/kg and 500 mg/kg) resulted in a significant decrease in the number of rearing was observed. Concerning the time spent at the central arena, the 125 mg/kg extract dose showed a significant (*p* < 0.01) increase in this parameter, while the 250 and 500 mg/kg doses showed a significant (*p* < 0.001) increase compared to the control group (Figure 4(c)).

### 3.6. Effect of the Aqueous Extract of *B. aegyptiaca* Fruit Pulp on Oxidative Stress Parameters

#### 3.6.1. Effect of the Aqueous Extract of *B. aegyptiaca* Fruit Pulp on Lipid Peroxidation

**Figure 5:**
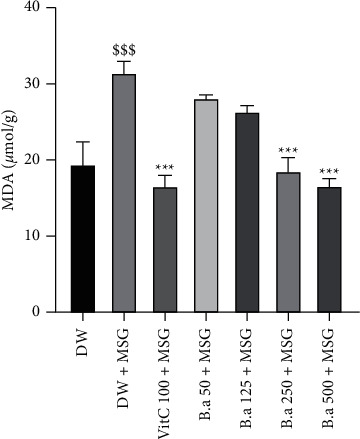
Effect of the aqueous extract of *B. aegyptiaca* fruit pulp on lipid peroxidation. Each column represents the mean ± SEM of six animals. MSG: monosodium glutamate, Vit C: vitamin C, DW: distilled water. ^*∗∗∗*^*p* < 0.01 versus negative group. ^$$$^*p* < 0.001 versus control group.

Administration of monosodium glutamate showed a significant (*p* < 0.001) increase in lipid peroxidation resulting in an increase in the concentration of MDA in brain homogenates compared to the normal control group. Administration of the extract (250 and 500 mg/kg) reversed the increment by significantly (*p* < 0.001) decreasing MDA concentration compared to the negative control group that received only monosodium glutamate (Figure 5).

#### 3.6.2. Effect of Aqueous Extract of *B. aegyptiaca* Fruit Pulp on Nitric Oxide Concentration in the Brain

**Figure 6:**
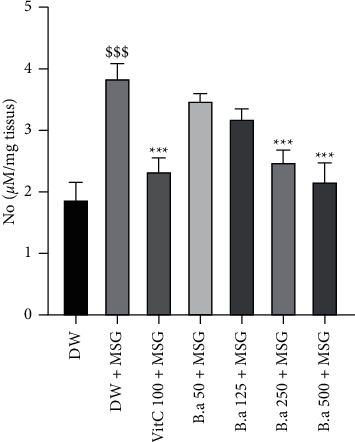
Effect of the aqueous extract of *B. aegyptiaca* fruit pulp on nitric oxide concentration in the brain. Each column represents the mean ± SEM of six animals. MSG: monosodium glutamate, Vit C: vitamin C, and DW: distilled water. ^*∗∗∗*^*p* < 0.01 versus negative group. ^$$$^*p* < 0.001 versus control group.

A significant (*p* < 0.001) increase in nitric oxide concentration in homogenates of the negative control group was observed after MSG administration compared to the normal control group. The extract (250 and 500 mg/kg) resulted in a significant (*p* < 0.001) decrease of this concentration compared to the negative control group (Figure 6).

#### 3.6.3. Effect of the Aqueous Extract of *B. aegyptiaca* Fruits on the Activity of Antioxidant Enzymes

**Figure 7:**
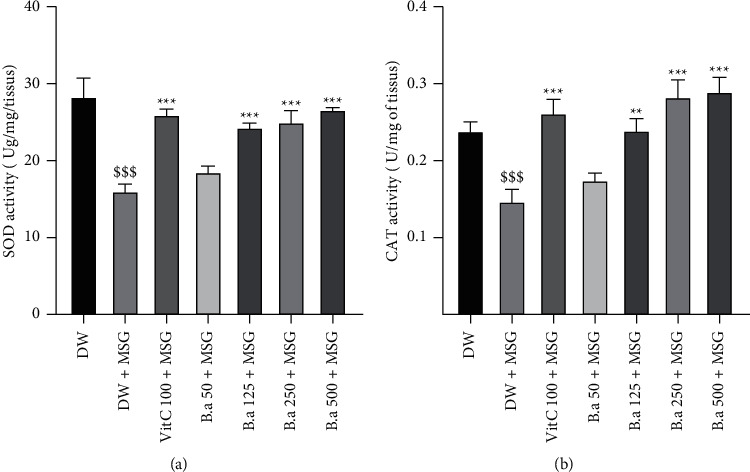
Effect of the aqueous extract of *B. aegyptiaca* fruits on the activity of antioxidant enzymes. Each column represents the mean ± SEM of six animals. (a) SOD activity and (b) CAT activity. MSG: monosodium glutamate, Vit C: vitamin C, and DW: distilled water. ^*∗∗*^*p* < 0.01 and ^*∗∗∗*^*p* < 0.001 versus negative group. ^$$$^*p* < 0.001 versus control group.

Figures 7(a) and 7(b) illustrate the effect of the aqueous extract of *B. aegyptiaca* fruit pulp on antioxidant enzymes activity. A significant decrease *p* < 0.001 in SOD and catalase activity was observed after the administration of MSG compared to the normal control group. Pretreatment with the extract showed a significant (*p* < 0.001) increase in the activity of these enzymes at 250 and 500 mg/kg doses compared to the negative control group receiving MSG.

### 3.7. Effect of Aqueous Extract of *B. aegyptiaca* Fruit Pulp on Cholinergic Transmission

**Figure 8:**
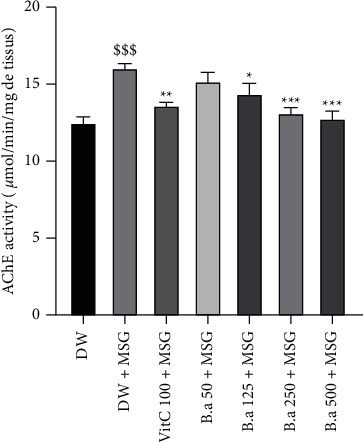
Effect of aqueous extract of *B. aegyptiaca* fruit pulp on cholinergic transmission. Each column represents the mean ± SEM of six animals. MSG: monosodium glutamate, Vit C: vitamin C, and DW: distilled water. ^*∗*^*p* < 0.05, ^*∗∗*^*p* < 0.01, and ^*∗∗∗*^*p* < 0.001 versus negative group. ^$$$^*p* < 0.05 versus control group.

Long-term administration of MSG significantly increased (*p* < 0.05) acetylcholinesterase activity in brain homogenates of the negative control group compared to the normal control group. However, the extract (250 and 500 mg/kg doses) significantly (*p* < 0.001) decreased acetylcholinesterase activity in brain homogenates compared to the negative control group (Figure 8).

### 3.8. Effect of Aqueous Extract of *B. aegyptiaca* Fruit Pulp on the Microarchitecture of the Hippocampus

**Figure 9:**
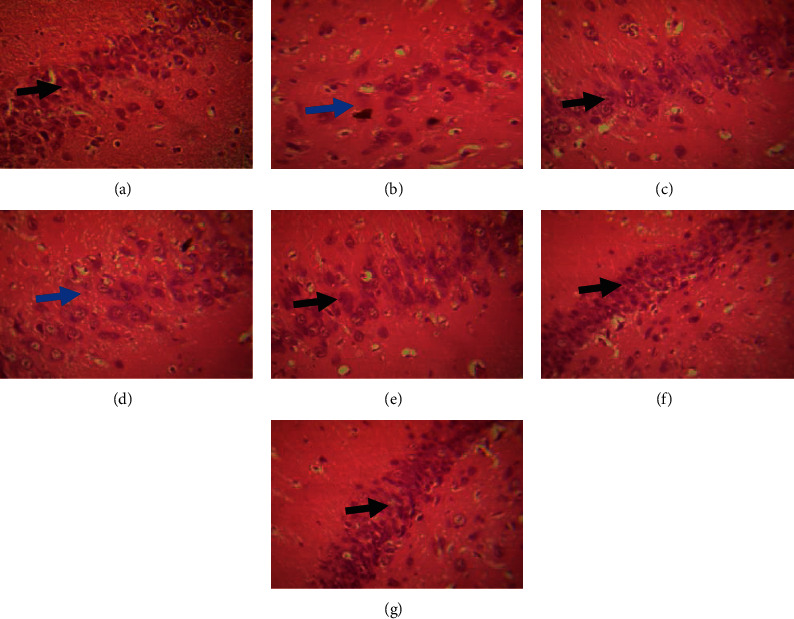
Effect of the extract of fruit pulp of *B aegyptiaca* (*B. a*) on the microarchitecture of the hippocampus: (a) control, (b) negative control, (c) positive control (vitamin C 100 mg/kg + MSG), (d) *B. a* 50 mg/kg + MSG, (e) *B. a* 125 mg/kg + MSG, (f) *B. a* 250 mg/kg + MSG, and (g) *B. a* 500 mg/kg + MSG. High-density cell on CA3 region, low-density cell on CA3 region, and MSG: monosodium glutamate.

Figure 9 below shows the microarchitecture of the hippocampal cornus ammoni (CA3). Histopathological analysis of the hippocampus shows that the administration of monosodium glutamate reduced the density of cell bodies of pyramidal neuronal cells (Figure 9(c)) in the CA3 region, materialized by a decrease in the cells density in this region compared to the normal control group. Animals treated with *B. aegyptiaca* extract exhibited a normal architecture of CA3 pyramidal cell layer with a high cell density characteristic of pyramidal neurons (Figures 9(d)–9(g)).

## 4. Discussion

The present study was undertaken to demonstrate the effect of the aqueous extract of *B. aegyptiaca* fruit pulp on memory loss induced by monosodium glutamate excitotoxicity in Swiss mice. The results of the phytochemicals study revealed the presence of polyphenols, flavonoids, and tanins. The values of total polyphenols, total flavonoids, and total tannins were higher than the values obtained by Koubala et al. [47] in some wild edible fruits from the far north region of Cameroon. The presence of those compounds in *B. aegyptica* pulp fruit could partly explain the pharmacological properties obtained in the present study. The object recognition, Y-maze, and Barnes maze were employed to assess memory and the open field for locomotion. MSG is a sodium salt of glutamic acid that has been reported to possess excitotoxic effects, most likely through neuronal damage [48, 49]. Its administration for a period of 30 days resulted in a significant decrease (*p* < 0.05) in the time taken to explore a novel object in the novel object recognition test, representing impairment in learning and memory [50]. The novel object recognition test evaluates different memory processes, such as acquisition, consolidation, and retrieval. The administration of 500 mg/kg *B. aegyptiaca* fruit pulp extract resulted in a significant (*p* < 0.001) increase in novel object exploration time as well as discrimination index compared to MSG solely treated group. The extract could restore long-term memory, therefore improving the learning and memory process. The Y-maze test is commonly used for screening new compounds with a stimulatory effect on memory in rodents. This test relies entirely on the innate ability of rodents to recognize the sequence of arm inputs (also known as spontaneous alternations) [51]. Thus, an animal with a memory deficit would not be able to perform a good alternation [52]. Administration of monosodium glutamate resulted in a significant (*p* < 0.01) decrease in the percentage of alternation compared to the normal control group reflecting a deterioration of short-term memory. These results were significantly (*p* < 0.05) reversed by pretreatment with aqueous extract of *B. aegyptiaca* fruit pulp at 250 and 500 mg/kg without any significant change in locomotion observed. This increase in the percentage of alternation observed could translate to an improvement in working memory (short-term memory) [53]. According to Lee and Goto [54], an increase in the percentage of spontaneous alternation is evidence of an improvement in the learning process and working memory in rodents. The relatively nonvariable number of lines crossed may suggest that the short-term memory enhancing the potential of the extract is not associated with any sedative effect. In order to verify the effect of the extract on reference memory and spatiotemporal memory, the Barnes maze test was performed. During the acquisition phase, the animal uses distal objects to locate the target hole. It thus develops a relative spatial memory based on the objects present around the maze, and its memory develops progressively with the different trials as in human interactions [55]. In this test, the administration of monodic glutamate to the negative control group showed a significant (*p* < 0.05) increase in the time taken to find the escape hole and a significant (*p* < 0.001) decrease in the direct search strategy compared to the normal control group. This reflects an alteration in memory capacity [56]. These results are in agreement with those obtained by Omogbiya et al. [57] who showed that the repeated administration of high doses of MSG altered spatial working memory. However, all doses of the extract resulted in a significant (*p* < 0.001) increase in the direct search strategy and a significant (*p* < 0.01) decrease in the time taken to find the target hole. This reflects the use of spatial cues to reach the target hole and thus an improvement in spatial memory [58]. In order to evaluate the impact of various substances on motor coordination, the open field test was performed. Results obtained disclosed a significant (*p* < 0.001) increase in the number of lines crossed (hyperlocomotion) and rearing. These results are in agreement with Quines et al. [59] findings, which found that MSG-induced hyperactivity in rats, portrayed by an increase in the frequency of rearing movements. This MSG-induced hyperactivity is thought to be related to the release of a glutamate-triggered Ca^2+^ influx and increased discharge [60]. However, administration of *B. aegyptiaca* fruit pulp extract attenuated MSG-induced hyperlocomotion and decreased rearing activity in mice, probably by modulating glutamate receptor function, suggesting its potential neuroprotective effect. Memory deficit in the glutamate-induced animal model is mediated by oxidative stress [61] evidenced by studies delineating oxidative stress-related neuronal damage and memory deficits [62]. The accumulation of ROS has been found in several chronic diseases, including AD suggesting that ROS may contribute to the pathogenesis of these diseases by inducing oxidative stress. ROS are constantly generated in redox processes during metabolism, which attacks biomolecules such as enzymes, lipids, proteins, DNA, and RNA resulting in irreversible damage to these important commodities of life [17]. Based on this, antioxidant treatment has therefore been suggested for the prevention and therapeutic approach to address memory loss [63]. In this study, treatment with monosodium glutamate led to a significant (*p* < 0.001) increase in NO and MDA concentration, associated with a significant (*p* < 0.001) decrease in antioxidant enzyme activity (SOD and CAT), a sign of oxidative stress, suggesting an increase in oxidative-nitrosative stress. In fact, nitric oxide is produced after hyperexcitability of NMDA receptors following the massive entry of Ca^2+^ into the post-synaptic element. This is likely to be linked to a free radical, the superoxide anion O^2−^ that forms peroxynitrite ONOO^−^ that cause neuronal damage or death by apoptosis (moderate stress) or necrosis (intense stress) [64]. This could account for the memory deficit in the behavioral tests observed above. Moreover, a significant (*p* < 0.001) decrease in MDA concentration and a significant (*p* < 0.001) increase in SOD and catalase activity was observed after the administration of *B. aegyptiaca* fruit pulp extract (250 and 500 mg/kg). The increase in activity of these two enzymes, associated with a decrease in the concentration of MDA, suggests that *B. aegyptiaca* fruit pulp extract possesses an antioxidant effect, which might thus be capable of reducing the risk of various degenerative diseases including AD, by prompting antioxidative activities.

According to Nampoothiri et al. [16], the administration of MSG leads to an alteration in the functioning of certain neurotransmitters, namely acetylcholinesterase (AChE). Inhibitions of AChE decrease the hydrolysis of ACh in the brain and increase cholinergic neurotransmissions, which might be helpful in treating mild to moderate levels of Alzheimer's disease [17]. In this work, administration of MSG resulted in a significant (*p* < 0.05) increase in AChE activity compared to the normal control group. Indeed, AChE is an enzyme that acts by degrading acetylcholine (a neurotransmitter strongly involved in the memorization process), which could partly justify learning and memory deficits observed in behavioral tests negative control groups. These effects were significant (*p* < 0.01) reversed by pretreatment with 500 mg/kg extract dose. The cholinesterase inhibitory activity of several medicinal plants has been reported in the literature [65]. Inhibition of acetylcholinesterase enzyme can restore cholinergic functions and allow more retention of acetylcholine in the brain, which is essential for enhancing cognitive functions, learning, and memory [17]. Thus, the positive effects of this extract on memory may be partially due to the modulation of the cholinergic system. From these facts, the extract could have anticholinesterase effects.

To verify these results, an analysis of microarchitecture of hippocampal CA3 regions was performed. The abundance of glutamatergic receptors found in the CA3 region is involved not only in the encoding of information but also in the storage and retrieval of information. Histopathological analysis of the hippocampus showed that chronic administration (30 days) of monosodium glutamate decreased the density of CA3 region pyramidal neuron cell bodies of cells, which may translate the occurrence of the processes of hippocampal neurodegeneration in negative control animals. The deficits in hippocampal neurons may be attributed to the redox imbalance, which altered hippocampal neurons, resulting in learning and memory deficits. However, pretreatment with *B. aegyptiaca* extract increased cell density (pyramidal cells) at the CA3 region, compared to pyramidal CA3 cell density of MSG solely treated mice. In effect, numerous projections of CA3 cells via axonal collaterals relay them to other CA3 neurons. The existence of this recurrent circuit makes the CA3 a strongly interconnected region of the hippocampus and thus important in the acquisition of information [66]. From all the above, the extract of *B. aegyptiaca* fruit pulp could prevent the loss of hippocampal cells and consequently could favor learning and memory processes. These pharmacological properties could be linked to the presence of certain compounds such as total flavonoids, polyphenols, and soluble tannins present in this extract [67, 68]. Hence, due to the presence of hydroxyl groups in their structures, phenolic compounds found in plants exhibit strong antioxidant potentials that are reported to display cholinesterase inhibition as well free radical scavenging effects. These phenolic components together with flavonoids are free could be responsible for the observed high antioxidant and anticholinesterase activities, thus justifying their protective effects in the occurrence of many neurodegenerative diseases such as AD [17].

## 5. Conclusion

The administration of monosodium glutamate caused neuronal damage, through oxidative and nitrosative stress and by altering acetylcholinesterase activity, leading to a deficit in the learning process and memory observed during behavioral tests. Disorders that may result could be corrected by the administration of the *B. aegyptiaca* fruits pulp extract, whose neuroprotective action is mediated by the antioxidant and anticholinergic potential of its biocomponents. This may justify its use in traditional medicine as an alternative therapy of cognitive impairment.

## Figures and Tables

**Table 1:** Mice distribution and treatment.

Groups	Treatment
Normal control group	Distilled water
Negative control group	Distilled water + MSG 4 mg/kg/day i.p.
Positive control group	Vit C 100 mg/kg + MSG 4 mg/kg/day i.p.
Test I group	*B*. *aegyptiaca* 50 per os + MSG 4 mg/kg/day i.p.
Test II group	*B*. *aegyptiaca* 125 per os + MSG 4 mg/kg/day i.p.
Test III group	*B*. *aegyptiaca* 250 per os + MSG 4 mg/kg/day i.p.
Test IV group	*B*. *aegyptiaca* 500 per os + MSG 4 mg/kg/day i.p.

**Table 2:** Polyphenolic compounds and antioxidant activity of *B. aegyptiaca* fruit pulp.

Phytochemicals	Mean ± standard deviation
Total polyphenols (mg EGA/100 g DW)	78.13 ± 0.70
Total flavonoids (mg EQ/100 g DW)	48.73 ± 0.05
Total tannins (mg EC/100 g DW)	19.23 ± 0.06

Note: mgEGA/100 g DW: milligram equivalent gallic acid per 100 g dry weight, mgEQ/100 g DW: milligram equivalent quercetin per 100 g dry weight, and mgEC/100 g D W: milligram equivalent catechin per 100 g dry weight.

## Data Availability

The data used to support the findings of this study are available from the corresponding author upon reasonable request.
